# Research Advances in Targeted Therapy for Heart Failure

**DOI:** 10.31083/j.rcm2410276

**Published:** 2023-10-07

**Authors:** Liu Miao, Yan-Li Liu

**Affiliations:** ^1^Department of Cardiology, Liuzhou People's Hospital, Affiliated of Guangxi Medical University, 545006 Liuzhou, Guangxi, China

**Keywords:** HF, targeted therapy, intestinal flora, signaling channels, mitochondrial autophagy, gene therapy

## Abstract

Cardiovascular disease is one of the major diseases threatening the health of Chinese residents, and the death rate has long been the highest on the disease spectrum in China. With the progress of population aging, the prevalence and mortality of cardiovascular diseases remain on the rise, and the current treatment effect on and prognosis of heart failure (HF) are not satisfactory. It is particularly important to explore the potential pathogenic mechanisms of HF and identify new therapeutic targets.

Studies have shown that the number of people suffering from cardiovascular diseases in China is currently approximately 330 million, and 8.9 million are heart failure patients [1]. Heart failure (abbreviated as HF) is a group of syndromes in which various structural or functional heart diseases lead to impaired ventricular filling and/or ejection function, and cardiac blood output cannot meet the metabolic needs of body tissues, with clinical manifestations of pulmonary and/or body circulation stasis and insufficient blood perfusion to organs and tissues. According to the ejection fraction, HF with reduced ejection fraction (HFrEF, left ventricular ejection fractions (LVEF) <40%), and HF with preserved ejection fraction (HFpEF, LVEF) can be classified, as well as fraction (HFpEF, LVEF ≥50%) and HF with midrange ejection fraction (HFmrEF, LVEF 40% to 49%). Drugs commonly used to treat HF include diuretics, angiotensin-converting enzyme inhibitors (ACEIs), β-blockers, aldosterone receptor antagonists, angiotensin domain receptor antagonists (ARBs), digitalis, ivabradine, vilisicam, sodium glucose cotransporter 2 (SGLT2) inhibitors, angiotensin receptor neprilysin inhibitor (ARNI), and Chinese herbal medicine. The current treatment effect and prognosis of HF are not very satisfactory. Therefore, it is particularly important to explore the potential pathogenic mechanisms of HF and discover new therapeutic targets.

## 1. Gut Flora and HF Targeted Therapy

The intestinal flora is a relatively complex ecosystem composed of a variety of intestinal microorganisms that reside in the human gut, they are large in number and diverse. Each microorganism has the capacity to produce hundreds of different known and unknown metabolites that act on the intestine itself [1]. Intestinal flora dysbiosis is a pathological lesion caused by the inhibition of sensitive intestinal bacteria due to age, diet, drug abuse, disease and other factors, following which uninhibited bacteria take the opportunity to multiply, thus causing flora dysbiosis and imbalance of intestinal flora metabolites, resulting in the disruption of normal physiological combinations. Studies have shown that intestinal flora dysbiosis is closely related to the development of HF, that metabolites of the intestinal flora, mainly short-chain fatty acids (SCFAs), trimethylamine-N-oxide (TMAO) and bile acid (BA), are involved in the development of HF through various metabolic pathways, and they affect the prognosis of HF [2]. Diet, drugs (antibiotics), probiotics and fecal microbiota transplantation (FMT) can alter the intestinal flora components and provide new therapeutic ideas for the treatment of HF.

Dietary interventions are the safest and most effective way to improve the intestinal flora. Marques *et al*. [3] administered a high-fiber diet or added acetate as a dietary intervention in mice using an HF model and observed that it led to an increase in SCFA levels, in turn resulting in blood pressure levels being effectively controlled and myocardial hypertrophy and myocardial fibrosis being reduced, thus improving cardiac function. The American College of Cardiology/American Heart Association guidelines formally adopted a strong recommendation, the Dietary Approaches to Stop Hypertension (DASH), which recommends a diet that is rich in fruits, vegetables, whole grains and low-fat dairy products, including meat, fish, poultry, nuts and legumes, and that limits sugary foods and beverages, red meat and legumes, and added fats. Several observational studies have been shown to reduce the incidence of HF [4, 5, 6]. The Mediterranean diet, mainly high in fruits, vegetables, legumes and whole grains and low in red/processed meats and refined carbohydrates, is beneficial in delaying cardiovascular disease (CVD) and HF. In fact, a systematic review and meta-analysis comparing randomized, controlled trials of the Mediterranean diet involving 10,950 people showed that the Mediterranean diet reduced the incidence of HF by 70% [7].

Antibiotic interventions, one of the most common and effective experimental interventions to regulate the intestinal flora in clinical practice. A variety of antibiotics have been shown to reduce the levels of inflammatory factors in the body, such as interleukin-1β (IL-1β), interleukin-6 (IL-6) and tumor necrosis factor-α (TNF-α). Studies have shown that rifaximin can promote the growth of beneficial intestinal bacteria, such as bifidobacteria and lactobacilli, through its bactericidal, antibacterial and anti-inflammatory effects [8]. Studies have reported that oral vancomycin reduces infarct size and improves postinfarct cardiac function in rats, and follow-up studies have shown that a mixture of streptomycin, neomycin, polymyxin B, and bacteriocin reduced myocardial infarct size and altered microbial-associated metabolites [9, 10]. However, their effectiveness in improving HF has yet to be further verified.

Probiotics are a group of intestinal physiological bacteria that live in mutually beneficial symbiosis with their hosts and contribute to their health [11]. Probiotics in clinical use include bacterial and fungal microorganisms, including Lactobacillus spp., Bifidobacterium spp. and *Saccharomyces boulardii * [12]. Probiotics can colonize the intestine, prevent the adherent colonization of pathogenic bacteria, regulate intestinal flora disorders, and improve the intestinal inflammatory response. It has been reported that treatment of rats with a drink containing *Lactobacillus plantarum* 299v 24 hours before coronary artery ligation reduced infarct size and improved left ventricular function [10, 13]. In another study, it was found that the administration of *Lactobacillus rhamnosus* GR-1 treatment significantly improved left ventricular hypertrophy and increased left ventricular ejection fraction in a rat model of acute myocardial infarction. In a randomized, double-blind, controlled study, *Saccharomyces boulardii* was beneficial in patients with HF, with short-term improvements in left ventricular ejection fraction and reductions in serum creatinine and inflammatory markers [14].

FMT is the transplantation of functional bacteria from healthy human feces into the patient’s gastrointestinal tract to improve the structure and composition of the intestinal flora, and it can reverse dysbiosis of the intestinal flora and re-establish its normal function in inflammatory bowel disease [15, 16], but the therapeutic role of FMT in other diseases is unclear. Standardization and optimization of FMT procedures are essential, including screening of suitable donors, development of noninvasive delivery methods (e.g., capsules), and identification of active ingredients to develop a rational and individualized therapeutic strategy. The ability of FMT to improve HF by restoring the diversity and function of the intestinal flora requires extensive experimental and clinical studies.

Chinese medicine interventions are rich in a variety of chemical components. In particular, Chinese medicine compounds not only contain alkaloids, polysaccharides, glycosides and other effective drug components, but they are also rich in vitamins, dietary fiber and other nutrients, with a variety of pharmacological effects. They can also provide intestinal nutrition, promote the repair of intestinal mucosal epithelial cells and the expression of tight junction proteins, regulate intestinal immunity, reduce intestinal inflammation, and facilitate the intestinal flora. Single Chinese medicines [17], Chinese medicine monomers and Chinese medicine compounds can take the intestinal flora as an effective target to play a pharmacological role in the prevention and treatment of chronic HF. For example, Bao Yuan Tang, which is a Proprietary Chinese Medicine, can improve the dysregulated intestinal flora in rats with isoproterenol-induced myocardial hypertrophy, thereby regulating the metabolism of short-chain fatty acids, bile acids, and amino acids involved in the intestinal flora so that the downstream pro-inflammatory, pro-oxidative, and pro-myocardial hypertrophy signaling pathways can be effectively inhibited, thus exerting its effects of lowering blood pressure and blood lipids, alleviating myocardial hypertrophy, and improving cardiac function [18].

## 2. Cytokines, Chemokines and Targeted Therapy for HF

A large body of evidence suggests that cytokines and chemokines are closely associated with HF. The proinflammatory cytokines TNF-α, IL-1, and IL-6 cytokine-induced growth factor synthesis could play a chronic fibrotic role, and it is an important factor in the pathogenesis of HFpEF. There is now experimental evidence that cytokine and chemokine targeting hold therapeutic promise for HF [19].

In the early 1990s, studies showed significantly elevated levels of circulating TNF-α in patients with HFrEF, along with increased TNF-α expression in the myocardium in experimental models of HF and in patients with cardiomyopathy [20, 21, 22]. There is evidence to support a pathogenic role of TNF-α in HF. Early clinical studies have shown attenuated cardiac dysfunction in patients receiving TNF-α antagonists [23]. The Randomized Enalapril Global Evaluation (RENEWAL) trial indicate that enalapril had no effect on the primary endpoint of death or hospitalization due to HF in patients with HFrEF [23]. The phase II anti-TNF-α trial in congestive HF examined the role of infliximab, a chimeric monoclonal anti-TNF-α antibody, in patients with HFrEF and showed adverse effects and increased all-cause mortality and HF hospitalization with infliximab compared with conventional therapy [24]. The above findings can be attributed to the role of TNF-α in promoting the development of heart failure and adverse myocardial remodeling. But TNF-α has been shown to have cardioprotective effects, so it could be a potential factor for targeting in HF, but more experimental studies are needed to explore and validate it.

There are 11 cytokine members and 10 receptors that make up the IL-1 family; of these, the most well-studied pathogenic mechanisms in the cardiovascular system are the IL-1α/IL-1β, IL-18 and IL-33/stromal cell 2 (ST2) axes. In mice, effective inhibition of the inflammatory response inhibits the myocardial remodeling process after myocardial infarction, which is entirely dependent on the genetic disruption of IL-1 signaling due to the deletion of the IL-1 signaling receptor interleukin-1 receptor 1 (IL1R1) [24]. The pharmacological targeting of the IL-1 cascade has also shown excellent protective effects in experimental animal models. Although the potential efficacy of IL-1 targeting in HF patients still needs to be confirmed in large clinical trials, there is considerable evidence that IL-1 may have a cardioprotective effect. In a study supporting the mechanistic role of IL-1β in the development of atherosclerotic thrombotic disease, a better prognosis was found in patients receiving the anti-IL-1β monoclonal antibody canakinumab (CANTOS study) [25] compared to those on standard therapy and with previous myocardial infarction and evidence of active inflammation (elevated high-sensitivity C-reactive protein (hsCRP)), compounded with a better prognosis. Risk of endpoint (non-fatal myocardial infarction, non-fatal stroke or death) by 15% compared to patients who did not option for. The pre-defined exploratory analysis of the CANTOS trial (an outcome study of post-anti-inflammatory thrombosis) data has increased our confidence in its use as increased IL-1β inhibition has significantly reduced HF hospitalization or HF-related mortality [26]. In treating a subgroup of HF patients with canakinumab, we also found the same positive results in both human and animal studies, even though the CANTOS trial was not designed solely to test the effectiveness of IL-1β-targeted therapy for HF. In patients with HF after myocardial infarction, another anti-Ll-1β antibody, gigocizumab, was found to have a protective effect on the myocardium in animal models, preventing death.

A number of cytokines have been implicated in the development of cardiovascular disease, including IL-11, leukemia inhibitory factor (LIF), cardiolipin-1 and tumor suppressor M. These are typical members of the gp130 family of cytokines, the most well-studied of which is IL-6. Tocilizumab significantly reduced circulating blood levels of N-terminal pro brain natriuretic peptide (NT-proBNP) in patients with rheumatoid arthritis without previous cardiovascular disease, indirectly demonstrating its protective effect in slowing the progression of HF [27]. In another trial, in patients with non-ST-segment elevation myocardial infarction (NSTEMI), a single effective dose of tocilizumab before coronary angiography was effective in suppressing not only systemic validation levels but also in reducing troponin T release, indirectly suggesting that suppression of inflammation is effective in protecting against myocardial necrosis. This experiment provides ample evidence of the protective effect of tocilizumab against ischemic myocardial injury in acute coronary syndromes [28]. The complexity of the effect of IL-6 on the inflammatory cascade is demonstrated by the opposite trend in serum C-reactive protein (CRP) and chemokine C-X-C motif chemokine ligand 10 and C-C motif chemokine ligand 4 (CXCL10 and CCL4) levels, which well illustrates the uncertainty of the classical and cross-signaling effects of cytokines in anti-IL-6 therapeutic agents. In this context, more studies and more definitive results are needed to confirm the use of IL-6 targeting for the treatment of HF, which remains a potential target for HF therapy and still holds good promise to be explored [29].

Chemotactic cytokines of 8–12 kDa, called chemokines, are responsible for regulating cellular localization as well as migration in development, homeostasis and inflammation in the body [30]. Of these, XC, CC, CXC and CX3C chemokines are the four most studied subfamilies. Among the inflammatory CC chemokines regarding HF, C-C motif chemokine ligand 4/major capsid protein-1 (CCL2/MCP-1) has the ability to inhibit cardiac remodeling and prevent persistent myocardial damage leading to ischemic necrosis, and has been suggested in numerous studies as a potential therapeutic target for HF [31, 32]. CCL2 in endothelial cells, vascular smooth muscle cells, monocytes and cardiomyocytes, whose expression is consistently and stably upregulated in animal models of HF with cardiac injury and cardiac remodeling, may be associated with Toll-like receptor (TLR)-mediated signaling activation, neurohumoral cascade responses or pro-inflammatory cytokine-mediated pathways [31, 33, 34]. As research continues to ascend, persistent high expression of CCL2 in HF animal experiments, with or without myocardial infarction, is closely associated with myocardial dysfunction, fibrosis, and ultimately cardiac remodeling. In contrast, in patients with HFrEF, peripheral blood CCL2 levels showed a significant positive correlation with heart failure symptoms and left heart systolic function [35]. In patients with advanced HF, peripheral blood CCL2 levels are also positively correlated with the occurrence of adverse cardiovascular events [36]. CCL2-driven pro-inflammatory signaling leads to a sustained increase in immune response in failing cardiomyocytes, resulting in further swelling, necrosis and thus an increased risk of death from heart failure; it has been reported that in animal models of myocardial infarction, blocking the CCL2/chemokine receptor 2 (CCR2) axis is effective in reduce infarct size and protect surviving myocardium [37, 38]. At the same time, CCL2 has a powerful fibrotic effect, causing dead myocardium to fill the gap left by myocardial death through fibrosis, which to some extent accelerates myocardial remodeling [39]. A number of researchers have also suggested that CCL2 has a direct myocardial damaging effect, rapidly leading to increased risk of death due to cardiac contractile dysfunction [40]. Therefore, effective inhibition of the CCL2/CCR2 axis is expected to be a target for the future treatment of HF.

## 3. Wnt Signaling Pathway and HF Targeted Therapy

Wnt is a cysteine-rich glycoprotein in the extracellular matrix (EMC) that plays a key role in a variety of pathological processes including neurodegeneration, osteoporosis, cancer, cardiac arrhythmias, and myocardial infarction. The Wnt (wingless/integrated) signaling pathway is a fundamental signaling pathway that regulates heart and vascular development and plays a critical role in the development of Frizzled (Fzd) receptors, low density lipoprotein (LDL) receptor related protein 5/6 (LRP5/6) receptors and the downstream signaling molecules glycogen synthase kinase 3β (GSK3β), and β-catenin, the dispersion protein Disheveled (Dsh), T-cell factor/lymph-like enhancer factor (Tcf/Lef), cell scaffold axis protein (Axin) and other receptor proteins involved in the Wnt signaling pathway [41].

Myocardial hypertrophy is a compensatory response of the heart. Some findings have confirmed that the Wnt/β-catenin signaling pathway plays an important role in the pathophysiology of myocardial hypertrophy. After cardiac injury, some conduction pathways that are active during the embryonic period, such as the Wnt/β-catenin signaling pathway, are reactivated to promote the progression of the myocardial remodeling process [42]. Malekar *et al*. [43] showed that the classical Wnt signaling pathway can be activated in Dsh-overexpressing transgenic mice and that Dsh-overexpressing mice develop a severe cardiac hypertrophy phenotype 3 months after birth. It has also been shown that the nonclassical Wnt pathway is also associated with cardiac hypertrophy [43, 44]. Current studies have clarified the role of the Wnt signaling pathway in pathological processes, such as myocardial hypertrophy, myocardial fibrosis, and wound healing after myocardial infarction, while its role in the pathological process of HF must still be elucidated by further studies. Myocardial hypertrophy and myocardial fibrosis, which are among the independent risk factors for HF, can therefore be considered potential targets for the treatment of HF.

Currently, some breakthrough research progress has been made in regulating the activity of the Wnt signaling pathway by targeted degradation techniques. Using targeted degradation technology to precisely modulate Wnt signaling pathway activity, which in turn can be used to treat heart diseases, such as HF, Yeguang Chen’s research team successfully synthesized axin-derived peptides based on binding to β-catenin using PROTAC (small molecule proteolytic targeting chimera) technology and found that two stapled peptides, SAHPA1 and xStAx, enhanced or weakened Wnt/β-catenin signaling, respectively, by coupling SAHPA1 or xStAx coupled to Von Hippel-Lindau (VHL) ligands to engineer PROTACs for efficient β-catenin protein degradation. The obtained xStAx-vhl maintained β-catenin degradation *in vivo* and strongly inhibited Wnt signaling in cancer cells and antigen presenting cell (APC)-/- like organs, and the results suggest that xStAxvhl highlights the potential of β-catenin degraders PROTACs as a novel class of anticancer drugs [45]. Further studies are needed to verify whether xStAx-vhl plays an important role in treating or delaying cardiac hypertrophy by degrading the β-catenin protein and downregulating the activity of the Wnt signaling pathway.

**Fig. 1.**
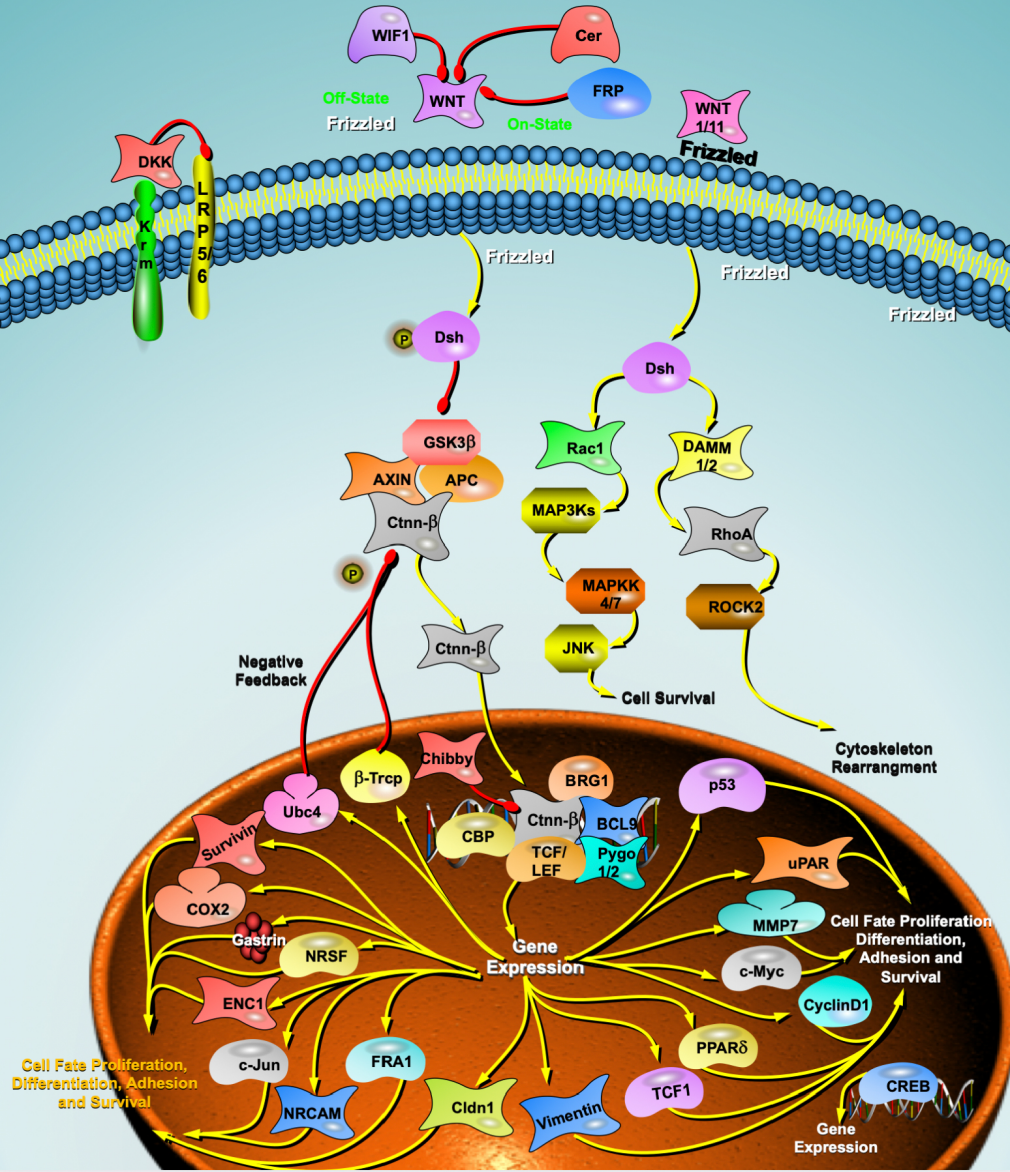
**Wnt signaling pathway and heart failure**. DKK, Dickkopf; WIF1, WNT inhibitory factor 1; BRG1, Brahma-related gene 1; Krm, Kremen; LRP5/6, lipoprotein receptor-related protein 5/6; FRP, Frizzled receptor proteins; Dsh, Dishevelled; GSK3β, glycogen synthase kinase 3β; APC, APC regulator of WNT signaling pathway; AXIN, axin protein; Ctnn-β, Catenin Beta; Rac1, Rac Family Small GTPase 1; DAMM1/2, Dishevelled Associated Activator Of Morphogenesis 1/2 MAPSKs, Mitogen‑activated protein kinase; MAPKK4/7, Mitogen-activated protein kinase kinase 4/7; JNK, c-Jun N-terminal kinase; ROCK2, Rho Associated Coiled-Coil Containing Protein Kinase 2; RhoA, Ras homolog family member A; cox2, Cyclooxygenase-2; ENC1, ectodermal-neural cortex 1; c-Jun, Transcription factor Jun; NRCAM, Neuronal Cell Adhesion Molecule; FRA1, Fos-related antigen1; Cldn1, Claudin 1; TCF1, T cell factor 1; PPARδ, peroxisome proliferator-activated receptors; CREB, cAMP response element binding protein; c-Myc, MYC proto-oncogene; MMP7, Matrix Metallopeptidase 7; uPAR, urokinase plasminogen activator surface receptor; p53, Tumor protein P53; Pygo1/2, Pygopus Family PHD Finger 1; BCL9, B cell lymphoma 9; TCF/LEF, T-cell factor/lymphoid enhancer factor; CBP, CREB binding protein; β-Tcrp, β-tranducin repeats containing protein; Ubc4, ubiquitin conjugating enzyme 4.

Other proteins in the Wnt signaling pathway can also be regulated by targeted degradation techniques. For example, Dsh proteins can regulate both classical and nonclassical Wnt signaling pathways, and to treat pathophysiological processes, such as cardiac hypertrophy, myocardial fibrosis, myocardial repair, and myocardial remodeling. In addition, the axin protein can be degraded by both the ubiquitin-proteasome pathway and the autophagy-lysosome pathway, and by designing specific PROTAC or autophagy-tethering compound (ATTEC) small molecule compounds, the function of the Wnt signaling pathway can also be precisely regulated (Fig. 1).

## 4. Mitochondrial Autophagy and Targeted Therapy for HF

Autophagy is the phagocytosis of eukaryotic cells by their own cytoplasmic proteins or organelles to achieve the metabolic needs of the cell itself and the renewal of some organelles through lysosomal degradation, the most important of which is mitochondrial autophagy in selective autophagy. Studies have shown that mitochondrial autophagy (mitophagy) is closely associated with HF [46, 47].

Mitochondrial autophagy is the process by which autophagic vesicles selectively wrap and transport damaged mitochondria to lysosomes for hydrolysis. This concept was first proposed by Lemasters in 2005 [48]. The current findings suggest two main types of mitochondrial autophagy mechanisms: ubiquitin dependent and non-ubiquitin dependent. Among them, ubiquitin-dependent mitochondrial autophagy includes PTEN-induced putative kinase 1 (PINK1)/parkin signaling pathway-mediated mitochondrial autophagy and non-parkin-dependent mitochondrial autophagy, while non-ubiquitin-dependent mitochondrial autophagy is directly mediated by mitochondrial autophagy receptors.

Song *et al*. [49] observed that parkin-mediated reduction in mitochondrial autophagy in cardiomyocytes from Mitofusin-2 (*MFN2*) gene mutant mice induced cardiac hypertrophy and HF. Chen *et al*. [50] found in a clinical study that PINK1 protein levels were significantly reduced and mitochondrial autophagy was inefficient in patients with advanced HF, whereas normal expression of PINK1 and parkin could attenuate myocardial cell injury, delay the progression of HF, and prolong patients’ lives.

It was found that the PINK1/parkin-mediated mitochondrial autophagy pathway is a potential target for the treatment of HF. In an experimental study of safranin, it was observed that parkin-mediated mitochondrial ubiquitination was significantly inhibited after knockdown of the PTEN-induced putative kinase 1 (*PINK1*) gene, counteracting the beneficial effects of safranin on HF, suggesting that safranin could promote PINK1/parkin signaling pathway-mediated mitochondrial autophagy for the treatment of HF [51]. Xiong* et al*. [52] found that overexpression of PINK1 promoted parkin translocation to the damaged mitochondrial outer membrane and phosphorylation, maintained cardiomyocyte homeostasis, and attenuated myocardial injury in a mouse model of angiotensin II-induced HF. In a tacrolimus (TAC)-induced mouse model of HF, Wang *et al*. [53] showed that high expression of AMP-activated protein kinase α2 (AMPKα2) in cardiomyocytes mediated by recombinant adeno-associated virus type 9 could activate the phosphorylation of Serrate284 and Serrate495 (Ser284 and Ser495) sites on PINK1, thereby increasing the role of the PINK1/parkin signaling pathway in mitochondrial autophagy and alleviating HF.

## 5. Noncoding RNA and HF Targeted Therapy

### 5.1 MicroRNAs

Noncoding RNAs (NCRs) are RNAs that do not encode proteins. MicroRNAs (miRNAs) are a class of single-stranded noncoding RNAs of approximately 22 nucleotides in length, and mature miRNAs contribute to the posttranscriptional degradation of target mRNAs by binding specifically to the $3^{\prime }$ untranslated region (UTR) of target mRNAs or by inhibiting the posttranscriptional degradation of target mRNAs [54].

A growing number of studies have shown that microRNAs (miRNAs) play an important role in cardiovascular disease and are involved in several pathophysiological processes associated with HF, such as myocardial remodeling, cardiac hypertrophy, myocardial fibrosis, apoptosis, and hypoxia [55, 56]. An increasing number of miRNAs have exhibited a dysregulated and predominantly upregulated pattern in the later stages of end-stage HF [57]. The etiology of HF (e.g., ischemic, aortic stenosis, or idiopathic cardiomyopathy) is associated with differentially expressed miRNA patterns [58]. Thus, miRNAs play an active role in the development and progression of HF.

In the plasma of hypertrophic cardiomyopathy (HCM) patients diagnosed with no symptoms of HF, miR-29a, the only miRNA associated with left ventricular hypertrophy and fibrosis, was found to be significantly upregulated [59]. In addition, miRNAs can be used to determine the prognosis of HF patients. Qiang *et al*. [60] examined miRNAs in endothelial progenitor cells (circulating from monocytes) from 106 HF patients and found that low levels of miR-126 were associated with cardiovascular death in patients with ischemic HF, while high levels of miR-508a-5p were associated with nonischemic HF patients. Additional studies have shown that decreases in miR-18a-5p and miR-652-3p during hospitalization for HF predicted 180-day mortality [61]. One meta-analysis examining the expression of circulating miRNAs and patient prognosis [62], which included four relevant articles assessing 19 circulating miRNAs in 867 patients, showed that low expression of miR-1, miR-423-5p, miR-126, miR-21, miR-23, miR-30d, miR-18a-5p, miR-16-5p, miR-18b-5p, miR-36b-5p, miR-206a-3p, miR-2313-3a-3p, and miR-423-128 was associated with significantly poorer overall survival in HF patients (*p* < 0.05). Among these molecules, miR-18a-5p, miR-18b-5p, miR-30d, miR-30e-5p, and miR-423-5p were strong biomarkers of HF prognosis.

It has also been shown that microRNAs can be used as biomarkers of response to HF treatment. For example, one animal study found [63] that plasma levels of miR-16, miR-20b, miR-93, miR-106b, miR-223 and miR-423-5p were elevated in rats with hypertension-induced HF compared to controls. Nie *et al*. [64] showed that miR-217 was highly expressed in the myocardial tissue of patients with chronic HF and exacerbated pressure load-induced cardiac dysfunction. These findings suggest that miR-217 plays an important role in myocardial hypertrophy and dysfunction and could be a therapeutic target for HF.

### 5.2 LncRNA

Long noncoding RNAs (lncRNAs) are a set of functional RNA molecules greater than 200 nucleotides in length that do not encode proteins and are generally present within longer coding genes, between coding genes, or in antisense to coding sequences [65]. LncRNAs are involved in cell differentiation, proliferation, apoptosis and autophagy, affecting the development and progression of various cardiovascular diseases, such as hypertension, atherosclerosis and HF. Liu *et al*. [66] showed that lncRNA H19 is highly expressed in the embryonic period, and its expression gradually decreases as individuals mature. The expression of lncRNA H19 decreases as individuals mature but increases again when vascular damage or cardiac insufficiency occurs. The results of another study observed a significant increase in H19 expression in patients with HF and in a mouse model of myocardial hypertrophy [67]. Han* et al*. [68] showed that high expression of H19 could be associated with disorders of lipid metabolism, and these findings suggest that the extent of HF might be understood by measuring H19 expression levels. In addition, studies have shown that the lncRNA long intergenic noncoding RNA predicting cardiac remodeling (LIPCAR) is upregulated early and downregulated later in the plasma of HF patients, and it has also been suggested that LIPCAR might be used as an HF biomarker [69]. Although the mechanisms of action of only a few lncRNAs have been elucidated, further systematic and comprehensive studies can be conducted to shed more light on the complex regulatory mechanisms and functional targets of lncRNAs, providing new ideas for the diagnosis and treatment of cardiovascular diseases and new targets for the development of new drugs.

## 6. $N^{6}$-Methyladenosine and HF Targeting Therapy

The types of RNA modifications include $N^{6}$-methyladenosine ($m^{6}$A), N1-methyladenosine, 5-methylcytosine, pseudouridine nucleoside, N6,$2^{\prime }$-O-dimethyladenosine, and $N^{7}$-methylguanosine. $m^{6}$A methylation is a methylation modification formed by the N at position 6 of adenine (A) catalyzed by methyltransferase, which occurs mainly in the highly conserved consensus motif of $5^{\prime }$-RRRACU-$3^{\prime }$ (R=A or G), in the beginning segment of the $3^{\prime }$ untranslated region of the mRNA molecule and near the termination codon. $m^{6}$A modifications are closely related to the development of cardiovascular diseases, including myocardial hypertrophy, HF, ischemic heart disease, aortic aneurysms, vascular calcification, and pulmonary hypertension.

Differentially $m^{6}$A-modified transcripts in HF patients are mainly involved in cardiac metabolism and signaling. Calmodulin 1 (Calm1) mRNA, a member of the calcium/calmodulin-dependent protein kinase II (CamKII) signaling pathway, was not altered during $m^{6}$A modification, whereas Calm1 protein expression was significantly reduced in failing heart tissue, suggesting that, in the development of HF, $m^{6}$A methylation affects calm1 translation, but not transcription, during the development of HF. $m^{6}$A-seq revealed differentially methylated transcripts of epigenetic proteins, transcription factors and upstream regulators of signaling pathways, suggesting that $m^{6}$A methylation is involved in the regulation of gene expression in HF [70]. Dorn *et al*. [71] showed that $m^{6}$A methylation levels are elevated by hypertrophic stimuli. The $m^{6}$A RNA methylation enzyme methyltransferase-like 3 (METTL3) plays an important and positive role in cardiomyocyte hypertrophy, and in an *in vitro* model, the growth tendency of cardiomyocyte hypertrophy is completely lost when METTL3 is inhibited, and no hypertrophy occurs, whereas overexpression of METTL3 promotes spontaneous and compensatory hypertrophy. In an *in vivo* model, cardiac-specific METTL3 knockout mice exhibited cardiac remodeling and HF, followed by dysregulation of cardiac homeostasis, while elevated levels of the $m^{6}$A RNA methyltransferase METTL3 led to cardiac hypertrophy.

Mathiyalagan* et al*. [72] found that obesity-associated protein (fat mass and obesity-association protein, FTO) is a demethylase that plays an important role in myocardial homeostasis and remodeling during cardiac contraction. FTO overexpression attenuates the ischemia-induced elevation of $m^{6}$A modification. $m^{6}$A modification is increased and FTO expression is significantly decreased in the infarct and peri-infarct regions of failing hearts compared to healthy heart tissue. FTO overexpression attenuated the ischemia-induced elevation of $m^{6}$A modifications. In FTO knockout mice, HF progressed more rapidly, with a lower ejection fraction and more severe cardiac dilatation, suggesting an integral role for FTO in HF. However, the molecular mechanism of action remains unclear, and many key issues must be further investigated to more deeply explain the regulatory mechanism of $m^{6}$A methylation in the development of the cardiovascular system, which could help to improve the diagnosis, treatment and prognostic judgment of the disease and provide a more scientific basis for the targeting of $m^{6}$A in the treatment of HF and other cardiovascular diseases in the future.

## 7. Other Targeted Therapies

### 7.1 ACE2-Ang1-7 Axis

The renin angiotensin system (RAS) plays a very important role in regulating normal physiology and the mechanisms of cardiovascular disease development. Angiotensin converting enzyme (ACE) is an important key enzyme of the classical RAS pathway angiotension converting enzyme-angiotensin II-type 1 (ACE-Ang II-AT1), which converts angiotensin (Ang) I to Ang II, and the increase in Ang II is an important part of the RAS involved in the development of cardiovascular diseases. Another RAS pathway, ACE2-Ang (1-7) MAS, exerts action antagonistic to the classical RAS pathway, and the two pathways are functionally antagonistic to each other, giving the RAS a dual effect [73]. Activation of the ACE2/Ang-(1-7) pathway effectively delays the progression of HF, and changes in the balance between ACE/Ang II and ACE2/Ang (1-7) play an important pathophysiological role in HF failure. Ang (1-7) is a cardioprotective peptide in the RAS that counteracts the cardiotoxic effects of Ang II and has protective effects against pathological cardiac remodeling and HF [74]. The ACE2-Ang (1-7) pathway, known as the second metabolic axis of the RAS, plays a crucial role in cardiovascular disease. Ang (1-7) plays a cardioprotective role and has the potential to contribute to HF treatment.

### 7.2 Advanced Glycation End Products

Advanced glycation end products (AGEs) are compounds produced by the nonenzymatic reaction of free amino groups in proteins, lipids, and nucleic acids with reducing sugars (e.g., glucose, fructose, pentose, etc.), i.e., the Maillard reaction, and they are classified as endogenous or exogenous according to their sources [75]. The exogenous pathway from food is the main source of AGEs in humans. Protein-bound AGEs have been most extensively studied, and AGEs affect downstream HF-related signals, including the proto-oncogene protein p21 (ras), stress-activated protein kinase (SAPK), the activator of transcription (STAT) pathway, and several other pathways, by coupling to cell membrane proteins and altering their structures or directly activating cell surface receptors [76, 77], promotes ventricular remodeling and myocardial injury during heart failure, and also participates in inflammation, oxidative stress and apoptosis of myocardial tissue and autophagy of cardiomyocytes. Gao *et al*. [78] found that Receptor of Advanced Glycation Endproducts (RAGE) activation in a mouse model caused excessive autophagic activity through activation of the RAGE-NF-κB (nuclear factor kappa B)/BNIP3 (BCL2/adenovirus E1B 19 kDa protein-interacting protein 3)/beclin1 signaling pathway, contributing to the apoptosis of cardiomyocytes to promote HF. In addition, the binding of AGEs to RAGE in the progression of HF can cause impairment of calcium metabolism, atherosclerosis, vasoconstriction, and myocardial fibrosis, thereby causing myocardial systolic-diastolic dysfunction. Studies have shown that AGEs bind to their receptors to promote the development of various cardiovascular diseases; therefore, by reducing AGEs or RAGEs and thus blocking the activation of AGE-related downstream cellular pathways, therapeutic or preventive effects on cardiovascular diseases can be achieved.

The most important exogenous source of AGEs is food, and the way in which food is cooked is the most important factor affecting the content of AGEs. The formation of AGEs is accelerated in high-temperature, long and deeply dry cooking methods (grilling, frying, deep-frying, etc.), so changes in cooking habits and methods can help to reduce the accumulation of AGEs in the body [79]. Toprak *et al*. [80] observed that the mechanism of the diastolic effect of alagebrium (ALT-711) on carotid arteries in healthy animals is not only to reduce the cross-linking of AGEs with collagen but that ALT-711 has the ability to improve the uptake and release of ${\mathrm{Ca}}^{2+}$ from the sarcoplasmic reticulum of cardiac myocytes. Some studies have shown that the angiotensin II receptor blockers telmisartan and losartan inhibit endogenous AGE production in cultured cells *in vitro * [81]. It follows that drugs can also antagonize AGEs.

### 7.3 Genetic Therapy

Due to the difficulties in targeting drugs to receptors and intracellular pathways, many years ago, researchers proposed cardiac gene therapy as an alternative therapeutic approach, whereby cell-targeted delivery of exogenous genes (transgenes) would produce “therapeutic” proteins that could compensate for pathological downregulation or counteract harmful molecular processes. The selection of appropriate targets and the availability of effective gene vectors are the key requirements for the success of this therapeutic approach.

A study published by Leiden’s research team in 1990 provided a history of cardiac gene therapy. In a landmark study, rat cardiomyocytes were transfected *in vivo* by direct injection of plasmid DNA containing the β-galactosidase gene in the left ventricular wall, and then β-galactosidase activity was found in the myocardium for up to 4 weeks [82]. In 2012, the CUPID (Calcium Up-Regulation by Percutaneous Administration of Gene Therapy in Cardiac Disease) study was the first clinical trial of cardiac gene therapy for HF. Replication-deficient adeno-associated virus type 1 (AAV1) was the vector selected to carry the Sarco/endoplasmic reticulum ${\mathrm{Ca}}^{2+}$-ATPase (SERCA2a) transgene. While the initial phase demonstrated safety and some beneficial effects, the final phase IIb CUPID trial was not successful. It involved 250 patients treated with intracoronary AAV1/SERCA2a or placebo. AAV1/SERCA2a treatment failed to prolong the time to the first endpoint event. Compared with placebo, AAV1/SERCA2a treatment had no significant effect on any endpoint, including New York Heart Association (NYHA) functional class, 6-minute walk test distance, or NT-proBNP levels [83, 84]. Despite the failure of the CUPID phase IIb trial, decades of preclinical studies have finally made gene therapy “palpable” in clinical cardiology.

As research on cardiac gene therapy has continued, a number of directions have been identified according to which good translational potential could exist. AC6 (adenylate cyclase) catalyzes the conversion of ATP into cyclic adenosine monophosphate (cAMP), a molecule essential for cardiac function; S100 calcium binding protein A1 (S100A1) is a protein that regulates sarcoplasmic reticulum ${\mathrm{Ca}}^{2+}$ cycling and mitochondrial function through interaction with ryanodine receptors, SERCA2 and mitochondrial F1-ATPase activity, with antihypertrophic, inotropic and antiarrhythmic effects and attenuating energy expenditure in HF [85]; SDF-1 (stromal cell-derived factor-1) has been shown to be essential in cardiac stem cell therapy; and a new frontier has also been proposed: gene therapy to stimulate cardiac regeneration. Other researchers have proposed cardiac stem cell therapy, among other therapies, as a targeted direction for the treatment of HF, but more studies and trials are needed to validate the roles of these therapeutic targets.

### 7.4 Chinese Herbal Treatment

Chinese medicine refers to natural drugs and their processed substitutes that are guided by the theory of Chinese medicine, have a unique theoretical system and application form, used to prevent and treat diseases and have rehabilitation and health care effects, mainly including plant drugs, animal drugs and mineral drugs [17]. Chinese medicine is divided into proprietary Chinese medicine and Chinese herbal medicine according to the processing undergone (Table 1). Chinese herbal medicines are rich in various chemical components, especially compounds that not only contain alkaloids, polysaccharides, glycosides and other effective drug components but are also rich in vitamins, dietary fiber and other nutrients, with a variety of pharmacological effects. The multitarget therapeutic effect of Chinese medicine can effectively avoid the adverse therapeutic effect caused by the defective therapeutic target and the weakened efficacy caused by the defective drug metabolism in the process of Western medicine treatment, and Chinese medicine can also effectively alleviate the clinical symptoms of patients with chronic HF, improve the prognosis of patients and improve quality of life. Chinese medicine monomers, single Chinese medicines and Chinese medicine compounds all have the characteristics of multichannel and multitarget therapy, which can regulate the body as a whole and affect the physiological functions of multiple systems of the body, so the targeted treatment of HF with Chinese medicine is also worthy of exploration [17, 86].

**Table 1.** **Proprietary Chinese medicines and herbal medicines available for the treatment of heart failure**.

Proprietary Chinese medicine	Herbal medicine
Baoyuan Tang, Danshen Yin, Shengmai San, Taohongsiwu Tang, Tinglidazaoxiefei Tang, Xuefuzhuyu Tang, Zhenwu Tang, Danqi Pill, Fufang danshen Dripping Pill, Shengmai Capsule, Qili Qiangxin Capsule, Qishen Yiqi Dripping Pill, Danhong Injection, Huangqi Injection, Shenmai Injection, Shenfu Injection, Shengmai Injection	Baishao, Baizhu, Bingpian, Chaihu, Chenpi, Chishao, Chuanxiong, Danggui, Danshen, Dazao, Fuling, Fupian, Fuzi, Gancao, Guizhi, Honghua, Hongshen, Huangqi, Jiangxiang, Jiegeng, Maidong, Maimendong, Niuxi, Renshen, Rougui, Sanqi, Shaoyao, Sharen, Shengdihuang, Shengjiang, Shudi, Tanxiang, Taoren, Tingli, Tinglizi, Wuweizi, Xiangjiapi, Yuzhu, Zexie, Zhiqiao

## 8. Conclusions

HF is a complex, multifactorial clinical syndrome with heterogeneity. Despite the disappointing results of current targeted therapy studies and targeted therapy being influenced by many factors, targeted therapy remains a promising and attractive direction for the treatment of HF. The potential factors leading to negative outcomes should be carefully analyzed, and further research and optimization should be promoted to discover new molecular targets with more significant therapeutic potential to exert cardioprotective effects and become new approaches to HF treatment.
